# Mental health of female foreign spouses in transnational marriages in southern Taiwan

**DOI:** 10.1186/1471-244X-11-4

**Published:** 2011-01-06

**Authors:** Bih-Ching Shu, For-Wey Lung, Ching-Hsien Chen

**Affiliations:** 1 Department of Nursing & Institute of Allied Health Sciences, National Cheng Kung University, Tainan, Taiwan; 2 Department of Psychiatry, Kaohsiung Armed Forces General Hospital, Kaohsiung, Taiwan; 3 Department of Neurology, Kaohsiung Medical University, Kaohsiung, Taiwan; 4 Department of Psychiatry, National Defense Medical Center, Taipei, Taiwan; 5 Calo Psychiatric Center, Pingtung County, Taiwan; 6 Department of Nursing, Meiho Institute of Technology, Pingtung County, Taiwan

## Abstract

**Background:**

The aim of this study was to investigate the mental health status, and the risk factors associated with mild psychiatric disorders, of female foreign spouses (from Vietnam, Indonesia, and mainland China) in southern Taiwan, and to understand the mental health needs of these women.

**Methods:**

One hundred and twenty nine participants were willing to participate in this study. All participants fulfilled all questionnaires which included demographic information, the Chinese Health Questionnaire (CHQ), the Eysenck Personality Questionnaire (EPQ), and the Mental Health Care Needs Questionnaire (MHCNQ).

**Results:**

By multiple linear regression, neuroticism characteristics (p = 0.000), the dimension of knowledge of the level of their own psychological disturbance (p = 0.001), dimension of friends assistance (p = 0.033), and dimension of religion comfort (p = 0.041) in mental health care needs could be used to predict possible mild psychiatric disorders. Furthermore, SEM model showed that Indonesian or Vietnamese spouses have more likely degree in mental health care needs (β = -0.24, *p *= 0.003), compared with Chinese ones. A higher level of neuroticism was associated with a greater likelihood of mild psychiatric disorder (β = 0.54, *p *< 0.001), and of mental health care needs (β = 0.21, *p *= 0.013). A higher degree of mental health care needs was related to a greater likelihood of mild psychiatric disorder (β = 0.14, *p *= 0.05).

**Conclusion:**

In conclusion, we have obtained a better understanding of the mental health status of female foreign spouses in transnational marriages, who face many difficulties. Indonesian or Vietnamese spouses tend to more likely degree in mental health care needs than Chinese spouses, and then indirectly influenced their mental health status. Some individuals with a neurotic personality are exposed to high risk and might suffer from mild psychiatric symptoms. The needs for psychological counseling and religion therapy were the first priority for these women, particularly the Indonesian and Vietnamese spouses. From these findings, we have a better understanding of how to assist these female foreign spouses in future.

## Background

As a result of globalization, the number of individuals who travel, work or study abroad is increasing, along with the number of transnational marriages. According to the United Nations [1], approximately half (49.6%) of the 200 million international migrants in 2005 were women. The UN has argued that globalization opens up a rather personal market of emotional relationships and marriages. Hence, the phenomenon of "mail-order brides" has become an important route for international migration, and the number of women from poor countries who have married men from more developed countries has increased. As a result, the number of marriage bureaus and matchmaking agencies has also risen. Recently, there has been a dramatic rise in the number of "mail-order brides" who have been provided through matchmaking agencies.

Today, cross-border marriages seem to be an alternative to poverty and starvation. Foreign brides usually come from countries such as Indonesia, the Philippines, Vietnam, and Thailand. Since the 1970s, such women have married men from the more affluent Western world, from countries that promise them a better life and a stable future, such as the United States, Canada, and Europe. In Asia, the recruitment of foreign brides has been increasing in Japan, Korea, Hong Kong, and Taiwan since the 1990s.

In 2006, one in every five marriages in Taiwan was transnational [2], and 89% of the foreign spouses involved were female. The majority of these female foreign spouses originated from countries in Southeastern Asia, including Vietnam and Indonesia. Such marriages were arranged predominantly through a commercial marriage agent [1]. The most common reason given for the decision to marry a foreign husband was economic stability (58%), because the marriage enables the woman to relocate and to obtain a higher social status and quality of life [3]. The other reasons included: to obtain a visa (16%), love (16%), curiosity about living in a foreign land (5%), an escape from family problems, and so on.

The impact of a transnational marriage on the relocated spouse includes changes in culture, language, role, and interpersonal interactions. According to Helman [4], culture is a set of guidelines that individuals inherit as members of a particular society. It tells them how to view the world, how to experience it emotionally, and how to behave in the world in relation to other people, to supernatural forces or gods, and to the natural environment. The context of culture comprises historical, economic, social, political, and geographical elements. Thus, the different cultural backgrounds of people from different countries might cause them to think, experience life, and behave differently. As a result, a move to another country is an important event for people, and it can alter their life dramatically.

Marriage, which is a big step in the life of an individual, also creates a new scenario in which the partners must adjust to their new roles. Female foreign spouses in Taiwan not only face the stress of immigration, but also have to cope with the cultural demands of their husband's family. In general, female foreign spouses go to more affluent countries, such as Japan, Hong Kong, Korea, and Taiwan, to gain a better life and a more stable future. These countries, including Taiwan, actively promote cross-border marriages and encourage the naturalization of foreign spouses in order to ensure a healthy productive line that will serve the needs and interests of neoliberal globalization [3].

Shu and colleagues interviewed 19 female foreign spouses and found them to have difficulties in adjustment to their new life in Taiwan [5]. The major themes raised regarding their living experience were: difficulty in finding a sense of belonging, children being the center of their life, and children being under pressure because of the insecurity and loneliness of their mother. Becoming a mother needs many more resources, and social networks are required to support the development of women emotionally, socially, psychologically, and culturally, while they develop their abilities as competent mothers. The need both to adjust to an alien culture and to serve as a mother causes foreign mothers to suffer from more pressures and conflicts than local mothers. Hence, the role of mental health services is very important in these populations, because mental health problems might result from these pressures [6-8].

Kuan suggested that the health and well-being of female foreign spouses is a critical issue [9]. Furthermore, the social, cultural, and economic adjustment required during the process of adaptation can be stressful. Immigration has been linked to mental health, but the nature of the association has changed over time. Early notions of immigration and mental health were built on the premise that people encounter difficulties and obstacles as they settle into a new society [10]. The cultural differences not only cause mental disability, but they also contribute to other problems, including difficulties in interpersonal relationships with partners and others [11]. Integration, and the adjustment to a new relationship and a new environment, can affect the health of an individual. Cultural differences, changes in life pattern, and feelings of hopelessness are risk factors for deviations from health. When immigrants have difficulty in adapting to new social and cultural norms, mental health problems such as depression, schizophrenia, and anxiety can be manifested [12,13].

However, despite the increasing visibility of transnational female foreign spouses in communities across the island of Taiwan, little is understood about their health, especially their mental health. The lack of health data about these women has become a critical issue as health care providers try to become responsive to the pressing needs of female foreign spouses who come from different cultures and potentially speak or read little or no Chinese. In order to help these female foreign spouses to adjust, it is necessary to understand better the experiences and concerns of these women, who bring their own cultural backgrounds, perceptions, and experiences into the homes of their husbands in Taiwan. Therefore, an understanding of the health care needs and mental health, as well as its related factors, of this specific group in Taiwan is an important issue for mental health nurses.

The specific aims of this study were: (1) to investigate the mental health status, as well as factors related to mental health, of female foreign spouses from Vietnam, Indonesia, and mainland China in transnational marriages, and (2) to understand the mental health care needs of these women.

## Methods

### Participants

The inclusion criteria for participants were: (1) individuals who had immigrated to Taiwan for marriage and whose nationalities were Indonesian, Vietnamese or Chinese; (2) age between 20 and 50 years; (3) the ability to comprehend Indonesian, Vietnamese, Mandarin, Taiwanese (Hokkien), Haka or English. The exclusion criterion was residence in their country of origin for more than 6 months of each year. The participants of the study were recruited from Pingtung County in southern Taiwan. The researcher began by contacting foreign mothers who brought their children for routine health checks in the local health center agencies. Afterwards, a larger number of qualifying foreign spouses were recruited through snowballing. Participants and their families were informed of the research procedures, the duration of the study, and that all the data provided would be anonymous. If they agreed to participate in the study, they gave written informed consent. The study was approved by the IRB of National Cheng Kung University Hospital. In order to ensure the validity of the data collected, the data collectors were trained to cultivate cultural sensitivity and culturally appropriate care ability. Three types of questionnaire were completed and the demographic details of the participants were collected at their home by a trained researcher.

### Outcome Measures

Four questionnaires were used to collect data.

#### 1. Demographic information

The information included the participant's age, marital status, level of education, family income, and attendance at a Chinese language program, and the ethnicity of their husband.

#### 2. The Eysenck Personality Questionnaire (EPQ)

The EPQ was developed initially by Eysenck and Eysenck [14]. It consists of four subscales: psychoticism, extraversion, neuroticism, and the lie scale. The Chinese version of the EPQ was modified by Lu [15]. It elicits 25 items and has two subscales, extraversion and neuroticism. A Cronbach's α of 0.90 and good validity have been demonstrated [15].

#### 3. The Chinese Health Questionnaire-12 (CHQ-12)

The present study used the Chinese Health Questionnaire (CHQ) to assess the psychological well-being of the participants. The CHQ [16] is a modified version of Goldberg's General Health Questionnaire (GHQ) [17]. The scale has a weighted classification, sensitivity, and specificity of 89%, 70%, and 95%, respectively [18]. The CHQ has a Cronbach's α of 0.84 [19]. There are four possible answers for each item: answers that correspond to "not at all" or "about as usual" are scored "0", whereas those that correspond to "more than usual" and "strong feeling" are scored 1. A total score of 12 points is possible. A higher score indicates that the psychological well-being of the individual is lower. The optimum cutoff point (the best compromise between high sensitivity and a low false-positive rate) is 3/4 from the Receiver Operating Characteristic (ROC) curves [20]. Cheng et al. [19] demonstrated an internal consistency of 0.79 for the CHQ.

#### 4. The Mental Health Care Needs Questionnaire (MHCNQ)

This was developed by Shu et al. [5] and is based on previous interviews of foreign spouses and a literature review. There are eight items with five-point Likert scale responses. These items assess the need for: (1) knowledge of the level of their own psychological disturbance, (2) health professionals or medical services, (3) psychological professional services, (4) family assistance, (5) assistance from friends, (6) community assistance, (7) comfort from religion, and (8) folk therapy (such as fortune telling). A higher score indicates a greater need for health care.

The four measures (demographic information, EPQ, CHQ, and MHCNO) were translated into Vietnamese and Indonesian using the following procedures. Firstly, two bilingual translators translated the Chinese versions of the four measures into Vietnamese or Indonesian. Then, the draft version was given to two Vietnamese and three Indonesian female foreign spouses for pre-testing, and has made some corrections based on their advice. The final translated and original versions were completed by two bilingual Vietnamese and Indonesian transnational spouses with equivalent results (100%). These procedures ensured the applicability of the four questionnaires in Vietnamese and Indonesian in order to avoid language and cultural bias.

### Statistical Analysis

The data were analyzed using the SPSS 17.0 for Windows software package (SPSS, Chicago, IL, USA). In addition, the cut-off points of all possible indexes were assessed using receiver operating characteristic (ROC) analysis, which is a method to measure the ability of an observer to identify a signal against a background of noise [21]. In addition, structural equation modeling (SEM) was performed using the AMOS 7.0 software package for Windows (SPSS, Chicago, IL, USA). SEM uses the chi-square fit test to investigate the overall fit of the model. P values greater than 0.05 and an adjusted goodness-of-fit (AGFI) greater than 0.90 with a root mean square error of approximation (RMSEA) of 0.05 (0.08) or less indicated that the model adequately described the observed data.

## Results

### Demographic Characteristics

In total, 129 female foreign spouses, 41 from Indonesia, 45 from Vietnam, and 43 from mainland China, completed the questionnaires. There were significant differences with respect to age (χ^2 ^= 29.4, *p *< 0.005) among the three groups. Seventy-eight percent of the Vietnamese women were aged between 20 and 30 years, the Indonesians were predominantly between 26 and 35 years, and the Chinese participants between 31 and 35 years. On average, the Vietnamese spouses were the youngest of the study group. The period of time since the women had immigrated to Taiwan was also significantly different among the three groups. A greater proportion of women from Indonesia and mainland China than from Vietnam had immigrated more than five years ago (76.2% and 82.9%, respectively, vs. 47.7%). Almost all the women from the three groups remained in their original marriage (*p *> 0.05). The level of education in the country of origin, attendance at a Chinese language program, ability to communicate, religion, and number of children were significantly different among the three groups (*p *< 0.001). The Indonesian women had, on average, received more years of education in their country of origin than those from Vietnam or China. In general, they could understand some Chinese and had a basic ability to speak the language. In contrast, more than half of the Vietnamese participants did not attend a Chinese language program. Spouses from mainland China, who spoke a similar language to that spoken in Taiwan, expressed no need to attend a Chinese program. The basic demographic information is shown in Table 1.

**Table 1:** Demographics of female foreign spouses in transnational marriages (n = 129)

	Vietnamese (n = 45) n (%)	Chinese (n = 43) n (%)	Indonesian (n = 41) n (%)	chi-square p value
**1. Age**				**29.41**
20-25	18 (40.0)	2 (4.7)	5 (12.8)	**0.000**
26-30	17 (37.8)	10 (23.3)	14 (35.9)	
31-35	4 (8.9)	18 (41.9)	10 (25.6)	
> 36	6 (13.3)	13 (30.2)	10 (25.6)	
**2. Time since immigration to Taiwan (years)**				**13.91**
< 5	23 (52.3)	10 (23.8)	7 (17.1)	**0.001**
> 5	21 (47.7)	32 (76.2)	34 (82.9)	
**3. Marital status**				1.15
Married	43 (95.6)	38 (90.5)	39 (95.1)	0.563
Single	2 (4.4)	4 (9.5)	2 (4.9)	
**4. Education level in Homeland**				**17.63**
< 6 years	17(37.8)	2(4.7)	14(34.1)	**0.001**
6~9 years	17(37.8)	17 (39.5)	14 (34.1)	
> 9 years	11(24.4)	24 (55.8)	13 (31.7)	
**5. Attendance at Chinese literacy program in Taiwan**				**36.47**
None	19 (42.2)	37 (92.5)	12 (29.3)	**0.000**
Literacy classes or supplement school	26 (57.8)	3 (7.5)	29 (70.7)	
**6. Language ability before move to Taiwan**				**63.81**
Only mother tone	41 (91.1)		2 (4.9)	**0.000**
Could speak Chinese, Haka or Taiwanese	4 (8.9)		39 (95.1)	
**7. Taiwanese or Haka ability (listening and speaking)**				**26.70**
Very good	21 (48.4)	39 (92.9)	34 (82.9)	**0.000**
Poor	19 (44.2)	3 (7.1)	4 (9.8)	
Neither	3 (7.0)	0 (0)	3 (7.3)	
**8. Mandarin ability (listening and speaking)**				0.352^a^
Very good	38 (86.4)		38 (92.7)	0.486
Poor	4 (13.6)		3 (7.3)	
**9. Religion**				**30.54**
None	7 (15.6)	21 (48.8)	0 (0.0)	**0.000**
Yes	38 (84.4)	22 (51.2)	40 (100.0)	
**10. Number of children**				**17.02**
None	2 (4.7)	5 (11.6)	4 (9.8)	**0.002**
1	27 (62.8)	15 (34.9)	8 (19.5)	
≧2	14 (32.6)	23 (53.5)	29 (70.7)	
**11. Husband's age**				4.82
< 35	9 (20.0)	6 (14.3)	3 (7.7)	0.777
35~39	10 (22.2)	9 (21.4)	8 (20.5)	
40~44	13 (28.9)	9 (21.4)	10 (25.6)	
45~49	9 (20.0)	11 (26.2)	12 (30.8)	
> 50	4 (8.9)	7 (16.7)	6 (15.4)	
**11. Husband's years of education**				6.74
Less than 6 years	3 (6.8)	0 (0)	4 (9.8)	0.150
6~9 years	16 (36.4)	10 (23.8)	14 (34.1)	
More than 9 years	25 (56.8)	32 (76.2)	23 (56.1)	
**12. Household income/monthly (NT dollars)**				7.44
≦20,000	15 (48.4)	11 (29.7)	8 (32.0)	0.115
20,000-40,000	10 (32.3)	20 (54.1)	16 (64.0)	
>40,000-70,000	6 (19.4)	6 (16.2)	1 (2.4)	

^a ^Fisher's exact test

### CHQ among the three groups

The Indonesian spouses attained the highest mean score on the CHQ (mean = 2.35, SD = 2.81). However, there were no statistically significant differences among the three groups by ANOVA (Table 2). If a cut-off score on the CHQ of 3 points was used, 35.4% of Indonesian spouses and 28.9% of Vietnamese spouses showed possible psychological distress; 27.5% of Indonesian spouses and 20.0% of Vietnamese spouses had a possible mild psychiatric disorder if a CHQ cut-off score of 4 points was used (Table 3).

**Table 2:** Mean CHQ scores of female foreign spouses in the three groups (n = 127)

	Mean (SD)	F	P
Vietnamese (n = 44)	1.78 (2.60)	2.625	0.076
Indonesian (n = 40)	2.35 (2.81)		
Mainland Chinese (n = 43)	1.16 (1.48)		

**Table 3:** The percentage of female foreign spouses with CHQ > = 3 or 4

	CHQ≧3 (Psychological distress)	CHQ≧4 (Mild psychiatric disorder)
Vietnamese (n = 44)	13/45 = 28.9%	9/45 = 20.0%
Indonesian (n = 40)	14/40 = 35.0%	11/40 = 27.5%
Chinese (n = 43)	6/43 = 14.0%	3/43 = 7.0%
Taiwanese native (n = 67, ref)		17.9%

### Health care needs among the three groups

With regard to the health care needs of the participants, although there was no significant difference among the three groups, the priority of their needs was almost the same. The need for family assistance was ranked at the top, followed by the need to know the level of their own psychological disturbance. The assistance of friends was ranked third and the need for professional health services fourth. However, the Chinese group showed the fewest needs, according to the MHCNQ questionnaire (Table 4).

**Table 4:** Priority of health care needs among the different groups

	Vietnamese (n = 44)	Indonesian (n = 40)	Chinese (n = 43)	Total	Rank
Family assistance	89	85	67	241	1
Friend assistance	65	81	61	207	2
Knowing the level of own psychological disturbance					
	65	83	41	189	3
Psychological professional service					
	63	46	32	141	4
Health professionals or medical service					
	57	53	25	135	5
Community assistance	43	62	25	130	6
Religion comfort	40	48	13	101	7
Folk therapy (such as fortune telling)					
	39	24	5	68	8
Total	461/44 = 10.50	401/40 = 10.03	269/43 = 6.26		

### Factors related to CHQ

Demographics (age, education level, communication skills, number of years in Taiwan and so on), personality characteristics, and mental health care needs were analyzed using multiple linear regression to investigate which factors were predictive of possible mild psychiatric disorders in female foreign spouses. The results showed that neuroticism characteristics (p = 0.000), the dimension of knowledge of the level of their own psychological disturbance (p = 0.001), dimension of friends assistance (p = 0.033), and dimension of religion comfort (p = 0.041) in mental health care needs could be used to predict the mental symptoms in female foreign spouses. The results are shown in Table 5. A higher level of neuroticism characteristics, higher degree of mental health care needs in knowledge of the level of their own psychological disturbance dimension, assistance from friends dimension and religion comfort dimension would coursed greater likelihood of mild psychiatric disorders.

**Table 5:** Parsimonious prediction model of mental health for female foreign spouses using total scores of CHQ

Variable	B	*P *value
Neuroticism	.31	.00
Knowing the level of own psychological disturbance	.54	.00
Friend assistance	-.32	.03
Religion comfort	.43	.04
Constant	-.60	.22

B: regression coefficient

### Receiver operating characteristic (ROC) analysis

The optimum cut-off points for the score of neuroticism obtained from the ROC curve were calculated to be 4/5 (area under curve = 0.689). The sensitivity was 58.8%, specificity 70.4%, positive predictive value (PPV) 88.2%, and the negative predictive value (NPV) was 31.1%.

### Structural equation model (SEM)

**Figure 1:**
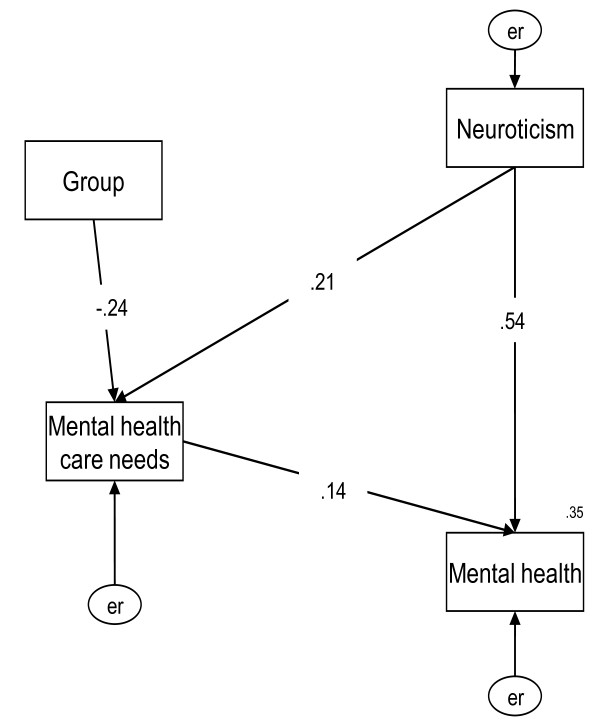
**Parsimonious model of mental health, mental health care needs and neuroticism among female foreign spouses**. Chi-square = 1.921; p = .383; df = 2; GFI = .993; AGFI = .963; RMSEA = .000. Dummy group: 1 = female foreign spouses whose nationalities were Chinese; 0 = foreign spouses whose nationalities were Indonesian or Vietnamese. Mental health = total score on CHQ; Mental health care needs = total score on MHCNQ; Neuroticism = total score for neuroticism; er = error.

The SEM was used to explore further the associations and interactions between neuroticism, mental health care needs and mental health among female foreign spouses. The parsimonious SEM resulted in a p value of 0.383, AGFI of 0.963 and RMSEA of 0.000, thus showing that the model accurately described the observed data (Figure 1). The dummy group was divided into female foreign spouses whose nationalities were Chinese, and whose nationalities were Indonesian or Vietnamese. Indonesian or Vietnamese female foreign spouses presented higher degree in mental health care needs (β = -0.24, *p *= 0.003), compared with Chinese participants, and then further indirectly influenced their mental health status. A higher level of neuroticism was associated with a greater likelihood of mild psychiatric disorder (β = 0.54, *p *< 0.001), and a higher degree of mental health care needs (β = 0.21, *p *= 0.013). Moreover, a higher degree of mental health care needs was related to a greater likelihood of mild psychiatric disorder (β = 0.14, *p *= 0.05). Particularly, the dimension of knowledge of the level of their own psychological disturbance (p = 0.01), dimension of psychological professional services (p = 0.05), dimension of religion comfort (p = 0.04), and dimension of folk therapy (p = 0.01) were main contributing factors of mental health care needs to increasing mild psychiatric disorders among groups (data not shown). The variance of mild psychiatric disorder was 35%.

## Discussion

This study provides valuable information on the mental health status of female foreign spouses in transnational marriages in Taiwan. Mental health has become a critical issue recently in the study of female foreign spouses. Spouses in transnational marriages are faced with the stresses associated with being away from their country of origin and with cross-cultural marriage. The present paper attempts to explore the mental health issues of these women, and to make general conclusions about their adjustment after immigration. This study also examined and identified various needs in relation to mental health. Furthermore, personality and mental health care needs factors were also found to be the potential threat to mental health.

Common problems, such as cultural adaptation, economic and language difficulties, and a lack of social support and ethnic identity, might mean that women in transnational marriages represent a high risk population with respect to mental health. In addition, factors such as effects on the self of family and social-cultural background might result in potential mental health problems when individuals become maladjusted [22]. Obviously, Chinese spouses tend to have better mental health status and less mental health care needs than Vietnamese or Indonesian spouses in the present study. There were around 20% and 27% of the Vietnamese and Indonesian spouses of this study, respectively, had a CHQ score ≥4, which can be compared with 18% in native Taiwanese women in our previous study [23]. This result indicated that these women in the current study had poorer mental health than those studied by Shu [23]. An important issue in social psychological research on mental health is the existence of stressful life circumstances that are rooted in the social role of the individual. Immigration is a stressful life experience that leads to cultural differences and conflicts, language barriers, and economic and societal changes, which in turn cause changes in interpersonal relationships, values and attitudes, and necessitates time to adjust. These changes can have negative effects on personal health [24,25]. Due to the similar social cultural background and language among China and Taiwan, hence, Chinese spouses suffer less likely psychological stress in living Taiwan, comparing with Vietnamese or Indonesian spouses. Vietnamese or Indonesian spouses also further need more assistance from friends and more comfort from religion than Chinese spouses.

Furthermore, it has been documented that many immigrant wives from Southeast Asia become pregnant soon after settling in Taiwan. Therefore, the difficulties involved in adapting to both a new marriage and a new culture are compounded by the challenges of giving birth and new motherhood. Most of the marriages of these female foreign spouses were arranged through matchmaking agencies. The relationship with the husband needs time to establish. In addition, because of the duty to continue the bloodline of the husband, these female foreign spouses not only face cultural differences but must also deal with the experience of giving birth to a baby. Although the female foreign spouses came to Taiwan for a better life, in general, the social-economic class of husbands involved in transnational marriages in Taiwan is low. There is also a lack of social support. Immigrants are often discriminated against, do not have close friends, and are unemployed; these factors also influence the indicators of depression and anxiety [26].

Good mental health implies that the individual has a stable personality and therefore can maintain a concordant relationship with the external environment. Mental health is affected by social structure, culture, and environment. The pursuit of the life task is an individual process and relates to personal beliefs and characteristics [27]. Our study showed that female foreign spouses with neuroticism were more likely to cause mental health care needs and to develop mild psychiatric symptoms than those without neuroticism. Research has shown that neuroticism is correlated with physical symptoms of mental health [28].

However, we found that the female foreign spouses did not identify their own health needs. Individuals commonly do not seek health care if they are unaware of their needs or do not identify the resources they could claim. From the responses of the participants, the need for assistance from the family was their first priority. The need for assistance from friends also played an important role. The stigma of acknowledging mental health problems poses a significant barrier to seeking help [29]. It seems that building a basic trusting relationship with such individuals is the first step. In an alien land, where the language, values, work atmosphere, and patterns of socialization are different from those in their own country, woman might become completely lost. Using further analysis of multiple linear regression, the dimensions of knowledge of the level of their own psychological disturbance, of friends assistance, and of religion comfort were potential threat factors in increasing mild psychiatric disorders. By SEM model, the findings also provided an evidence that higher degree of mental health care needs was related to greater likelihood of mild psychiatric disorder, especially the dimensions of knowledge of the level of their own psychological disturbance, of psychological professional services, of religion comfort, and of folk therapy. As description in the previous paragraph, Vietnamese or Indonesian spouses tend to have more likely mental health care needs than Chinese spouses, and then further indirectly caused more psychological stress, even leaded more mild psychiatric disorders. From the results of this study, we have a better understanding of how to assist these female foreign spouses, particularly focus on the psychological counseling and religion therapy in the Indonesian and Vietnamese spouses.

The psychosocial health of female foreign spouses is compromised not only as a consequence of having to cope with the usual demands of new motherhood and physical health problems but also as a result of loneliness caused by isolation and a lack of social support [30,31].

In general, physical health is given much greater priority than mental health. These female foreign spouses and their families did not perceive the stress of transnational marriage and its effects on mental health to be a problem. They considered it to be a self-inflicted abnormal behavior to seek attention. Thus, an examination of the use of mental health services by this population is necessary.

There are some limitations of this study. Firstly, the design lacked a control population of native Taiwanese women. In order to reduce the effect of this limitation, we used a previous study as a reference for comparison. Secondly, we used a convenience sample. A variety of women in transnational marriages have complicated reasons for coming to Taiwan. Therefore, it is difficult to make generalizations from our results. Thirdly, the cross-sectional design might not manifest the true situation comprehensively. It might be necessary to follow up the process of adaptation, adjustment, and incorporation into society regularly.

### Implication

Knowledge of these issues will help mental health nurses and other health workers to deal effectively with female foreign spouses in transnational marriages who originate from Indonesia or Vietnam. Immigrant women, especially foreign spouses, encounter socio-economic, political, and cultural problems that are imbedded in the societies they originated from and joined. These issues include problems related to prejudice, domestic violence, and discrimination. How female foreign spouses cope with and adapt to their new life and environment is important. Many immigrant women in Taiwan are isolated because they do not speak Mandarin, especially who originate from Indonesia or Vietnam. This creates a barrier owing to lack of communication between the woman and the external environment, and might reduce access to health care. An opportunity to learn the local language or dialect would help them to navigate their way around and enable them to socialize outside the house.

## Conclusions

According to Helman [4], "culture is a set of guidelines which individuals inherit as members of a particular society, and which tells them how to view the world, how to experience it emotionally, and how to behave in it in relation to other people, to supernatural forces or gods, and to the natural environment" (p. 2). The context of culture comprises historical, economic, social, political, and geographical elements. Thus, people from different countries can have different cultural backgrounds that cause them to think, experience life, and behave differently. As a result, a move to another country is an important event for people, and can alter their life dramatically.

Transnational marriages have become a popular social phenomenon. Several issues arise in association with transnational marriage. The need to adjust to an alien culture causes female foreign spouses to suffer from more pressures and conflicts than local mothers. A previous study showed that foreign-born mothers, especially those who could not speak the local language, had higher levels of depression and anxiety than native-born mothers [32]. In the same study, the researchers also found that children of native-born mothers performed better in developmental evaluations than those of foreign-born mothers.

As a result of "mail-order" marriages and unskilled migration, the above study reported an unmet need for social and economic security to ensure safe maternal practices and to reduce the challenges that face immigrant women in Southeast Asia countries. This paper seeks to fill that gap in documentation. A review article concluded that the transition to motherhood is an overwhelming experience. At present, "mail-order" brides from Southeast Asia make up the majority of transnational marriages. Furthermore, it has been documented that immigrant wives from Southeast Asia generally become pregnant soon after settling in Taiwan. The challenges of new motherhood are compounded, therefore, by the difficulties involved in adapting to both a new marriage and a new culture.

## Competing interests

The authors declare that they have no competing interests.

## Authors' contributions

All authors contributed to the design of the study. BC design and collected the data for the study. FW analyzed the data and wrote the first draft of the manuscript. CH collected the data and set up the database for the study. All authors have revised the manuscript and have approved the final manuscript.

## Pre-publication history

The pre-publication history for this paper can be accessed here:

http://www.biomedcentral.com/1471-244X/11/4/prepub
